# Pseudopterosin A-D Modulates Dendritic Cell Activation in Skin Sensitization

**DOI:** 10.3390/md23060245

**Published:** 2025-06-10

**Authors:** Johanna Maria Hölken, Katja Friedrich, Russel Kerr, Nicole Elisabeth Teusch

**Affiliations:** 1 Institute of Pharmaceutical Biology and Biotechnology, Heinrich-Heine University Düsseldorf, Universitätsstraße 1, 40225 Düsseldorf, Germany; johanna.hoelken@hhu.de (J.M.H.);; 2 Department of Chemistry, Department of Biomedical Sciences, Atlantic Veterinary College, University of Prince Edward Island, Charlottetown, PE C1A 4P3, Canada

**Keywords:** pseudopterosin, *Antillogorgia elisabethae*, skin, dermal dendritic cells, NiSO_4_, dexamethasone, CD54, CD86, IL-8, NF-kB

## Abstract

This study investigates the anti-inflammatory effects of the marine diterpene glycosides pseudopterosin A-D (PsA-D) in mitigating nickel sulfate (NiSO_4_)-induced skin sensitization. In dermal dendritic cell (DDC) surrogates, PsA-D pre-treatment significantly reduced NiSO_4_-induced upregulation of key activation surface markers, cluster of differentiation (CD)54 (~1.2-fold), and CD86 (~1.6-fold). Additionally, PsA-D inhibited the NiSO_4_-induced activation of the nuclear factor kappa-light-chain-enhancer of activated B cells (NF-κB) pathway by suppressing inhibitor of kappa B alpha (IκBα) degradation. Furthermore, PsA-D suppressed inflammatory responses by inhibiting the NiSO_4_-induced secretion of pro-inflammatory cytokines, including interleukin (IL)-8 (~6.8-fold), IL-6 (~2.2-fold), and IL-1β (~5.3-fold). In a full-thickness human skin model incorporating DDC surrogates, topical application of PsA-D effectively attenuated NiSO_4_-induced mRNA expression of IL-8 (~2.1-fold), IL-6 (~2.6-fold), and IL-1β (~2.2-fold), along with the key inflammatory mediators cyclooxygenase-2 (COX-2) (~3.5-fold) and NOD-like receptor family pyrin domain-containing 3 (NLRP3) (~2.1-fold). Overall, PsA-D demonstrated comparable efficacy to dexamethasone, a benchmark corticosteroid, providing a promising therapeutic alternative to corticosteroids for the treatment of skin sensitization and allergic contact dermatitis. However, to maximize PsA-D’s therapeutic potential, future studies on optimizing the bioavailability and formulation of PsA-D are required.

## 1. Introduction

In recent decades, marine-derived natural products have emerged as a source of bioactive compounds with promising therapeutic potential. Among them, pseudopterosins—a class of diterpene glycosides isolated from the octocoral *Antillogorgia elisabethae* (formerly *Pseudopterogorgia elisabethae*), which is endemic at depths of 15 to 35 m in the Caribbean Sea—have attracted considerable interest due to their distinctive chemical structures and biological activities [1]. Initially identified in the early 1980s, pseudopterosins have been the focus of extensive research exploring their pharmaceutical and biomedical applications [2,3]. To date, 31 chemical derivatives of pseudopterosin have been identified [4] from different *Pseudopterogorgia* specimens collected in the Bahamas [2,5], Bermuda [6], Florida Keys [7], and Colombia [1,8]. Distinct pseudopterosin derivates have been reported to possess a variety of promising biological activities, including anti-microbial [9,10], anti-inflammatory [7,9,11], analgesic [2,3,11], and anti-cancer activity [12,13,14,15]. In terms of skin disorders, pseudopterosins have been described as potent compounds for wound healing, increasing angiogenesis, reepithelization, and enhanced wound repair [16,17,18,19]. Associated with this, the semi-synthetic PsA methyl ether derivate (methopterosin/OAS1000) has completed Phase I and II clinical trials for wound healing [20]. However, these trials were discontinued due to the lack of strong therapeutic effects and drugability issues. Interestingly, pseudopterosins have been used as biological active ingredients in the cosmetic skin care product line Resilience by Estée Lauder since 1995 to prevent or to reduce skin irritation [21,22]. However, despite its many years of use in a market product, the potential underlying mechanisms of action of pseudopterosin in mitigating skin sensitization-related immune responses remains elusive.

Skin sensitization can be triggered by exposure to low-molecular-weight chemicals or metal ions such as nickel sulfate (NiSO_4_), leading to the development of an allergic reaction. This process can result in allergic contact dermatitis (ACD), a delayed-type hypersensitivity reaction characterized by inflammation upon re-exposure to the sensitizing agent [23]. Keratinocytes recognize allergens through oxidative stress-sensitive pathways including the Kelch-like ECH-associated protein 1 (Keap1), transcription factor nuclear factor erythroid 2 (Nrf2), or the NOD-like receptor family pyrin domain-containing 3 (NLRP-3) inflammasome pathway [24,25], resulting in the release of pro-inflammatory cytokines such as interleukin (IL)-1 and IL-8 [26,27]. These cytokines facilitate the recruitment and activation of immune cells including dendritic cells (DCs) and enhance the inflammatory response [28,29]. Concurrently, DCs start to internalize the allergens, undergo maturation processes, and migrate towards the draining lymph nodes to present the processed antigens to naïve T cells, finally leading to antigen-specific immune activation [30,31]. Maturation and migration of DCs is associated with increased expression of adhesion molecules such as cluster of differentiation (CD)54 and co-stimulatory molecules such as CD80 and CD86, which enhance antigen presentation to T cells and contribute to the initiation of the immune response [28,32]. A pivotal regulator of this inflammatory cascade is the nuclear factor kappa-light-chain-enhancer of activated B cells (NF-κB) pathway, which governs the transcription of numerous inflammatory mediators including CD54, CD86, and IL-8 [33,34]. 

The current standard of care for ACD involves the topical use of synthetic corticosteroids [23], such as dexamethasone, which are known for their immunosuppressive effects in various inflammatory disorders [35]. However, as long-term corticosteroid use is associated with adverse effects, including skin atrophy, barrier dysfunction, and systemic immunosuppression [36,37], there is a demand for alternative and potentially more gentle anti-inflammatory agents with improved safety profiles. Due to their distinct biological effects, further research into pseudopterosins appears promising in this context. First identified by Look et al. in 1982, PsA demonstrated stronger anti-inflammatory effects than indomethacin, an analgesic from the group of nonsteroidal anti-inflammatory drugs, against phorbol-myristate acetate (PMA)-induced inflammation in mice when applied topically (effective doses: 8.93 × 10⁻^4^ M vs. 40 mM) [3]. Mayer et al. confirmed these effects for PsA and PsE, with PsA showing higher potency [11]. Notably, systemic administration produced similar outcomes, ruling out non-specific irritation such as localized vasoconstriction [11]. Semi-synthetic PsA derivatives, like the O-Me C-glycoside, also resulted in dose-dependent anti-inflammatory activity [38], and Flachsmann et al. confirmed sustained anti-inflammatory activity across various structural modifications of the core structure [39].

Mechanistic investigations have linked the anti-inflammatory effects of pseudopterosins to the modulation of the arachidonic acid (AA) cascade. Pseudopterosin A (PsA) was shown to inhibit pancreatic phospholipase A2 (PLA2) and selectively target human neutrophil-PLA2, potentially via interactions involving its glycoside moiety [3,40]. In murine peritoneal macrophages, PsA significantly reduced the production of prostaglandin E_2_ (PGE_2_) and leukotriene C_4_ (LTC_4_) [3]. These findings were corroborated by in vivo experiments, where pseudopterosin E (PsE) suppressed the release of key eicosanoids following zymosan stimulation [11]. Comparative studies further demonstrated that PsA strongly inhibited (80%) PGE_2_ (IC_50_ = 4 µM) and LTC_4_ (IC_50_ = 1 µM) production in vitro, while PsE exhibited only minor effects (<40%) [11]. This discrepancy between the in vitro and in vivo efficacy of PsE suggests a requirement for metabolic activation to achieve significant inhibition of eicosanoid release [11]. Additionally, methopterosin (o-methyl-pseudopterosin), a methylated derivative of PsA, was identified as an inhibitor of cyclooxygenase-1 (COX-1). Although inhibition of both COX-1 and 5-lipoxygenase (5-LOX) was observed, significant effects were only achieved at relatively high concentrations (>500 μM and 253 μM, respectively) [11]. In human embryonic palate mesenchymal (HEPM) cells, methopterosin also reduced IL-1β-induced PGE_2_ production [41]. 

Beyond the AA pathway, our previous study shows that PsA-D reduced lipopolysaccharide (LPS)- as well as tumor necrosis factor (TNF)-α-induced NF-κB activation in the triple negative breast cancer cell line (TNBC) MDA-MB-231 and in the human monocytic cell line THP-1, by inhibiting the phosphorylation of p65 and IκBα in both cell lines, leading to a reduced expression of the pro-inflammatory cytokines and target genes of NF-κB: TNF-α, IL-6, and monocyte chemotactic protein (MCP)-1 [12]. In addition, PsA-D significantly reduced IL-6, IL-8, and TNF-α expression in peripheral blood mononuclear cells (PBMCs) [13]. Notably, PsA-D treatment led to translocation of the glucocorticoid receptor alpha (GRα). Consequently, knocking down the glucocorticoid receptor, PsA-D lost the ability to reduce the cytokine expression, indicating that PsA-D inhibits NF-κB through activation of the glucocorticoid receptor [12,13].

However, the high lipophilicity and poor aqueous solubility of pseudopterosins have significantly limited their investigation and application in biological systems. Although their amphiphilic structure suggests potential solubility, they remain largely water-insoluble, limiting their bioavailability. This limitation in solubility may contribute, at least partially, to the observed differences between the in vitro and in vivo efficacy of some pseudopterosin derivates. Approaches such as the synthesis of pseudopterosin succinate salts and alternative formulation strategies have been pursued to enhance their solubility and bioavailability but have been only partially successful [17]. Conversely, the high lipophilicity of PsA-D may offer advantages for topical application by facilitating its passage through the stratum corneum and promoting local retention within the epidermal layers. This could support sustained anti-inflammatory effects while minimizing systemic absorption—an important consideration in treating localized skin inflammation such as in skin sensitization and allergic contact dermatitis.

**Figure 1:**
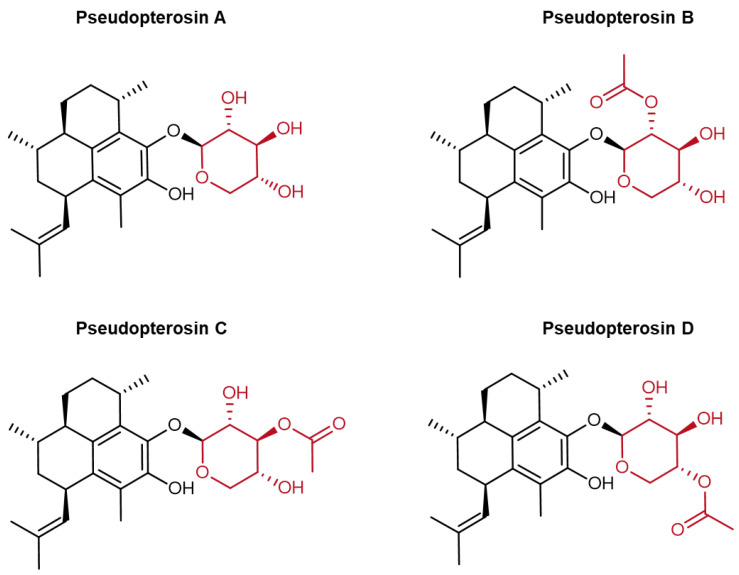
Chemical structures of pseudopterosins **A**, **B**, **C**, and **D** isolated from *A. elisabethae*. The aglycone amphilectane skeleton is depicted in black with the respective glycosylation at the C-10 carbon in red. Chemical structures have been illustrated with ChemDraw (Version 23.1.2).

Collectively, these mechanisms position pseudopterosins and particularly PsA-D (Figure 1) as promising candidates for the topical treatment of inflammatory skin disorders, warranting further investigation into their therapeutic potential. 

This study is the first to examine the anti-inflammatory effects of PsA-D on NiSO_4_-induced inflammation in isolated dermal dendritic cells (DDCs) as well as in an engineered full-thickness human skin model. In DDCs, we assessed the impact of PsA-D on NiSO_4_-induced activation by measuring CD54 and CD86 expression; inhibitor protein kappa B alpha (IκBα) degradation; and cytokine secretion including IL-8, IL-6, and IL-1β. Utilizing our established immune-competent skin model [42], we evaluated the effects of PsA-D on NiSO_4_-induced activation by analyzing mRNA levels of CD54, CD86, IL-8, IL-6, IL-1β, NLRP3, and COX-2, thereby exploring its potential as a novel therapy for skin sensitization and allergic skin inflammation.

## 2. Results

**Figure A1:**
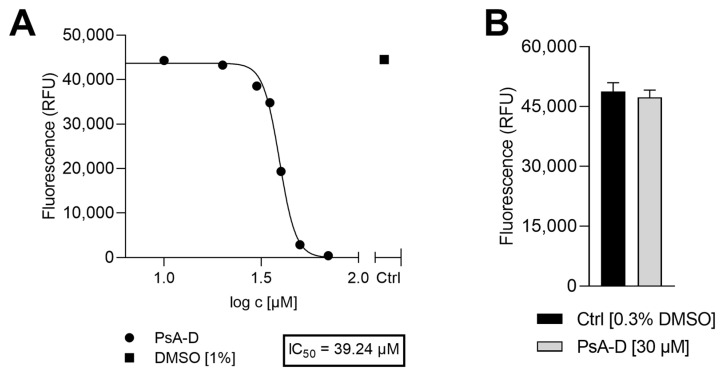
Determination of the cytotoxicity and half-maximal inhibitory concentration (IC_50_) of pseudopterosin A-D (PsA-D) (**A**) on DDC surrogates. Cells were seeded in triplicate for each treatment concentration as well as for the solvent control [1% DMSO]. PsA-D was applied in decreasing concentrations [70 μM, 50 μM, 40 μM, 35 μM, 30 μM, 20 μM, 10 μM, 1 μM] for 24 h. Cell viability was determined according to the PrestoBlue assay and is depicted in relative fluorescence units (RFUs). (**A**) depicts one experiment (with three technical replicates) out of three independent experiments. (**B**) depicts the cell viability of the DDC-incorporated skin models after topical exposure to PsA-D for 24 h.

To identify suitable treatment concentrations for PsA-D, the potential impact on cell viability of DDC surrogates was evaluated 24 h after incubation with a range of PsA-D concentrations (1–70 µM). As shown in Appendix A Figure A1A, a concentration-dependent reduction in cell viability was observed with an estimated half-maximal inhibitory concentration (IC_50_) of 39 µM and, at lower concentrations ≤20 µM, cell viability remained comparable to the control (1% DMSO), suggesting that PsA-D is well tolerated at these concentrations within the selected time window of 24 h (Appendix A Figure A1A). To further determine a putative cytotoxic concentration of PsA-D in the engineered human skin model, we topically treated the DDC-containing skin with 30 µM PsA-D for 24 h. Notably, as shown in Appendix A Figure A1B, a topical administration of PsA-D up to a concentration of 30 µM did not affect cell viability compared to the skin models treated with the solvent control (0.3% DMSO).

### 2.1. Pseudopterosin A-D Reduces NiSO_4_-Induced Upregulation of DC Activation Markers

Although pseudopterosins are known for their anti-inflammatory properties, their impact on distinct immune cells including dendritic cells (DCs) has been studied to a limited extent. Pseudopterosins have been shown to inhibit human and murine neutrophil degranulation and migration towards inflammatory stimuli, thus reducing the tissue damage associated with excessive immune responses [43]. Additionally, pseudopterosin A has been shown to reduce zymosan-induced secretion of prostaglandin E2 (PGE2) and leukotriene C4 (LTC4) in murine peritoneal macrophages [11], suggesting a potential modulatory effect on macrophage activation and polarization. Despite these established immunomodulatory activities, the impact of pseudopterosins on dendritic cells (DCs) has not yet been investigated. Given the central role of DCs in initiating and regulating immune responses, we were keen to investigate the potential of pseudopterosins on DC activation in skin sensitization. In particular, we sought to assess whether pseudopterosins A-D modulate the upregulation of CD54 (ICAM-1) and CD86 on dermal dendritic cells, two critical surface markers involved in antigen presentation and T cell activation. For this, DDC surrogates were pre-treated with PsA-D [20 µM] or the synthetic corticosteroid dexamethasone [1 µM] for 1 h prior to exposure to the sensitizer NiSO_4_ [380 µM] for 23 h. In untreated control cells, baseline surface marker expression of CD54 was ~78%. Upon treatment with NiSO_4_ [380 μM], CD54 expression increased significantly to ~97%, reflecting robust dendritic cell activation. Pre-treatment with PsA-D [20 μM] reduced CD54 expression to 84%, a similar level of suppression observed with dexamethasone [1 μM], which lowered CD54 expression to 86%. These results indicate that PsA-D effectively counteracts NiSO_4_-induced upregulation of CD54. 

**Figure 2:**
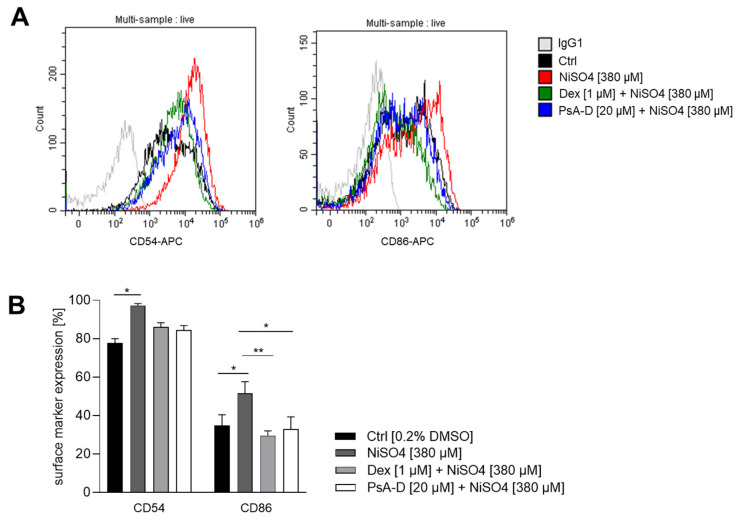
Pseudoperosin (Ps)A-D reduces the nickel sulfate (NiSO_4_) induced upregulation of DC activation markers CD54 and CD86 in a similar manner as dexamethasone. THP-1-derived dermal dendritic cell surrogates (DDCs) were seeded with 1 × 10^6^ cells/mL into a 24-well plate. Cells were pre-treated with dexamethasone [1 µM] or PsA-D [20 µM] for 1 h, followed by NiSO_4_ [380 µM] treatment for 23 h. Surface marker analysis was performed via flow cytometry and is depicted as histograms (**A**) and percentage of positive cells (**B**). Error bars indicate the standard errors of the mean (*n* = 3 independent experiments with * = *p* ≤ 0.05 and ** = *p* ≤ 0.01).

Baseline CD86 expression in control cells was ~35%. As expected for a skin sensitizer, NiSO_4_ [380 µM] treatment elevated CD86 expression to ~52%, confirming the pro-inflammatory effect. Notably, pre-treatment with PsA-D [20 µM] for 1 h reduced CD86 expression to ~33%, comparable to the inhibition achieved with 1 µM of dexamethasone, which lowered CD86 expression to ~30%, demonstrating the ability of PsA-D to mitigate NiSO_4_-induced activation of CD86 in dermal dendritic cells (Figure 2). 

In conclusion, PsA-D pre-treatment reduced the NiSO_4_-induced upregulation of DC activation markers CD54 and CD86 on DDCs in a similar manner as the synthetic corticosteroid dexamethasone. Thus, PsA-D effectively reduces DDC activation, thereby potentially affecting T cell priming (via reduced CD54 and CD86 expression). Hence, PsA_-_D could be a promising candidate for treating skin sensitization and allergic contact dermatitis

### 2.2. Pseudopterosin A-D Blocks the Activation of the NF-κB Pathway Induced by NiSO_4_

In our previous study, we demonstrated that the sensitizer NiSO_4_ activated DDC surrogates by inducing the degradation of IkBα, which in turn activated the NF-kB signaling pathway [42]. Based on our findings and those from other studies, we hypothesized that the NF-kB pathway could serve as an effective target for skin sensitization. Notably, research on the mechanism of action of PsA-D has identified PsA-D as a potent inhibitor of NF-κB signaling in triple-negative breast cancer (TNBC) [12]. Consequently, we sought to investigate whether PsA-D could inhibit the NiSO_4_-induced degradation of IkBα in the DDC surrogates.

**Figure 3:**
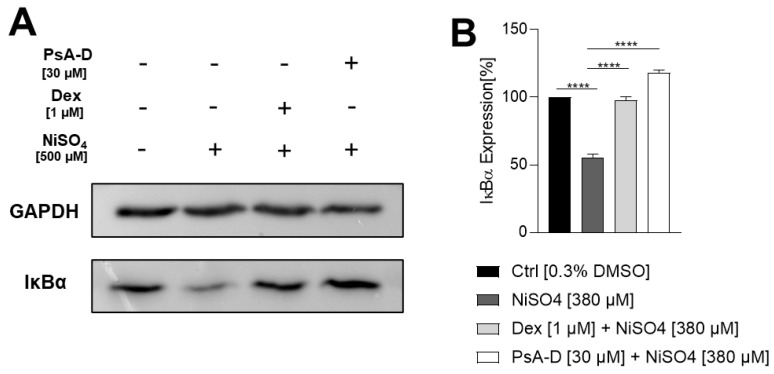
Pseudopterosin A-D blocks the NiSO_4_-induced degradation of IκBα in a similar manner as dexamethasone. DDC surrogates were seeded with 1 × 10^6^ cells/mL into a 24-well plate. Cells were pre-treated with dexamethasone [1 µM] or PsA-D [30 µM] for 1 h, followed by NiSO_4_ [500 µM] treatment for 1 h. (**A**) depicts one representative blot of three independent experiments. GAPDH served as loading control. (**B**) shows the quantification of image bands normalized to the solvent control. Error bars indicate the standard error of the mean (*n* = 3 independent experiments with **** = *p* ≤ 0.0001).

Western blot analysis (Figure 3A) revealed a considerable reduction in IκBα levels upon NiSO_4_ treatment compared to the solvent control, which was mitigated by both dexamethasone and PsA-D. GAPDH served as a loading control. Quantitative densitometry analysis (Figure 3B) quantified this effect: the solvent control was set at 100%, while NiSO_4_ treatment reduced IκBα levels to 56%. Pre-treatment with dexamethasone or PsA-D restored IκBα levels (Dex: 98%; PsA-D: 118%) relative to the control, demonstrating statistically significant protection.

### 2.3. Pseudopterosin A-D Suppresses NiSO_4_-Induced Secretion of Iinflammatory Cytokines

Previous studies had demonstrated that PsA-D significantly reduced the release of pro-inflammatory cytokines, including TNF-α, IL-6, and MCP-1, in LPS- and TNF-α-stimulated THP-1 monocytes and MDA-MB-231 breast cancer cells, an effect linked to the stabilization of IκBα and inhibition of p65 phosphorylation [12]. Based on these findings and considering that our prior work demonstrated a significant NiSO_4_-induced upregulation of cytokines including IL-8, IL-6, and IL-1β [42], we aimed to determine whether pre-treatment with PsA-D could similarly attenuate this inflammatory response.

**Figure 4:**
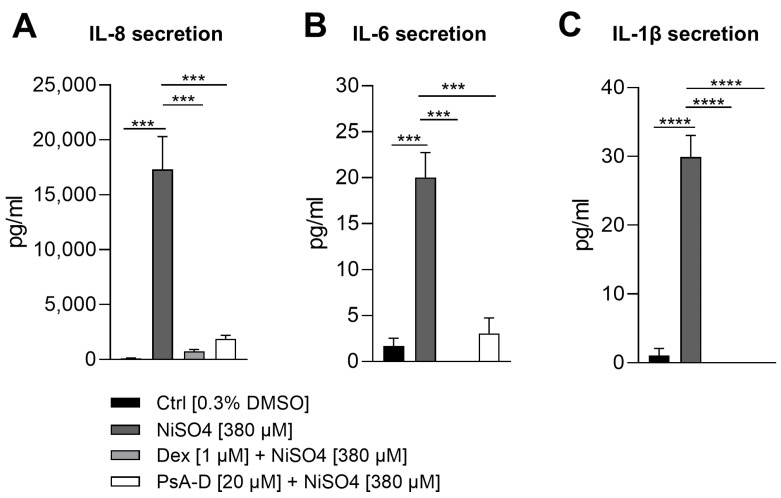
Pseudoperosin A-D suppresses the NiSO_4_-induced secretion of (pro-)inflammatory cytokines IL-8 (**A**), IL-6 (**B**), and IL-1β (**C**) in a similar manner as dexamethasone. DDC surrogates were seeded with 1 × 10^6^ cells/mL into a 24-well plate. Cells were pre-treated with dexamethasone [1 µM] or PsA-D [20 µM] for 1 h, followed by NiSO_4_ [380 µM] treatment for 23 h. Cytokine concentrations in the supernatant were determined using a Cytometric Bead Array Assay. Error bars indicate the standard errors of the mean (*n* = 3 independent experiments with *** = *p* ≤ 0.001, and **** = *p* ≤ 0.0001).

NiSO_4_ stimulation resulted in a substantial increase in IL-8 secretion, with levels rising from 118 pg/mL in control conditions to 17,303 pg/mL, representing a 146.6-fold induction. As expected, pre-treatment with dexamethasone significantly attenuated this response, reducing IL-8 levels to 735 pg/mL (a 23.5-fold decrease relative to NiSO_4_ alone). PsA-D also suppressed IL-8 secretion, yielding a concentration of 1873 pg/mL, corresponding to a 9.2-fold reduction compared to NiSO_4_ treatment alone (Figure 4A). Baseline IL-6 levels in the control group were measured at 1.7 pg/mL, whereas NiSO_4_ exposure elevated secretion to 20 pg/mL, representing an 11.8-fold increase. Dexamethasone completely abolished IL-6 secretion, reducing it to undetectable levels (0 pg/mL). Similarly, PsA-D pre-treatment led to a marked decrease in IL-6 secretion to 3 pg/mL, representing an approximate 6.7-fold reduction compared to NiSO_4_ alone (Figure 4B). In terms of IL_-_1β secretion, cells exposed to the solvent control exhibited minimal IL-1β secretion (1 pg/mL), while NiSO_4_ treatment induced a significant increase to 30 pg/mL, representing a 30-fold induction. Notably, both dexamethasone and PsA-D completely suppressed IL-1β secretion, reducing cytokine levels to undetectable amounts (0 pg/mL) (Figure 4C).

### 2.4. Pseudopterosin A-D Mitigates NiSO_4_-Induced DDC Activation and Inflammatory Signaling in 3D Human Engineered Skin

**Figure 5:**
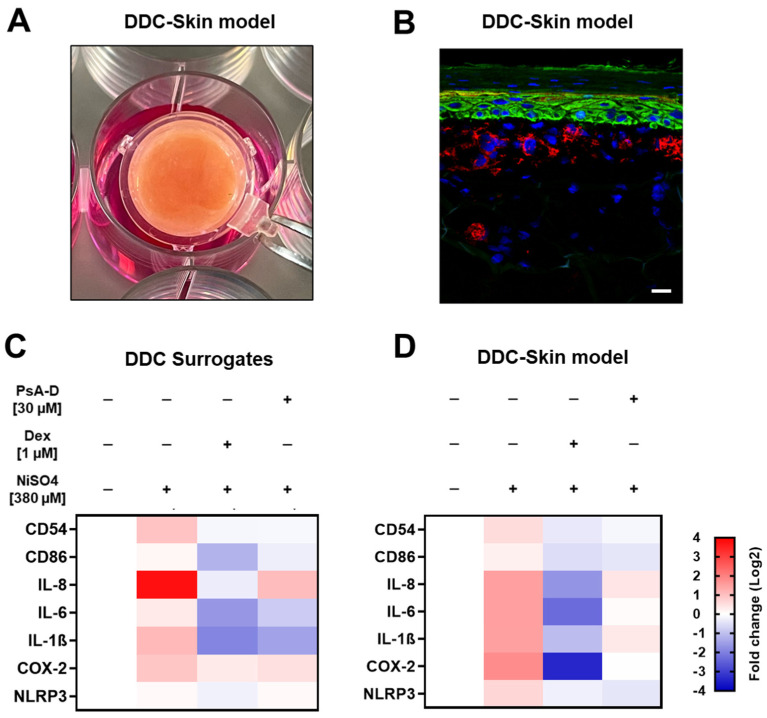
PsA-D reduces NiSO_4_-induced DC activation and inflammation markers on isolated DDC surrogates and DDC-skin models. (**A**) The image illustrates our immune competent full-thickness skin model on ALI day 10. The skin model exhibits a diameter of 1.4 cm, a height of 0.3 cm, and a surface area of 1.5 cm^2^. (**B**) Immunofluorescence staining of the immune competent full-thickness skin model with incorporated DDC surrogates (red). Keratinocytes were stained with cytokeratin 5 (green signal). DDC surrogates were stained with CD45 (red signal). Nuclei were stained with DAPI (blue signal). Scale bar = 20 µm. (**C**) DDC surrogates were seeded with 1 × 10^6^ cells/mL into a 24-well plate. Cells were pre-treated with dexamethasone [1 µM] or PsA-D [20 µM] for 1 h, followed by NiSO_4_ [380 µM] treatment for 6 h. (**D**) Skin models incorporated with DDC surrogates were pre-treated topically with dexamethasone [1 µM] or PsAD [30 µM] for 1 h, followed by NiSO_4_ [380 µM] treatment for 6 h. Skin models were mechanically and enzymatically dissociated and RNA was extracted for cDNA synthesis for RT-qPCR. Results are depicted as fold of change (Log2) compared to the solvent control [0.03% DMSO] and normalized to the expression of the housekeeping gene [GAPDH].

To investigate the effects of PsA-D on NiSO_4_-induced DDC activation and inflammation, we assessed mRNA expression levels of key activation markers, such as CD54 and CD86, along with inflammatory cytokines including IL-8, IL-6, and IL-1β, and inflammatory mediators like COX-2 and NLRP3. These markers were analyzed in both isolated DC surrogates (Figure 5C, Table 1) and a human full-thickness skin model containing DDC surrogates (Figure 5D, Table 2) using RT-qPCR. 

In isolated DDC surrogates (Figure 5C, Table 1), exposure to NiSO_4_ resulted in an upregulation of the mRNA expression levels of dendritic cell activation markers, specifically of CD54 (~1.9-fold) and CD86 (~1.1-fold). Pre-treatment with dexamethasone [1 µM] for 1 h significantly suppressed CD54 and CD86 expression by 2.1-fold and 2.8-fold, respectively. Similarly, pre-treatment with PsA-D [20 µM] for 1 h resulted in a significant reduction of CD54 (~2.1-fold) and CD86 (~1.4-fold) below baseline mRNA expression (1.0). 

Additionally, NiSO_4_ [380 µM] exposure of DDCs for 6 h significantly upregulated the mRNA expression levels of key inflammatory cytokines such as IL-8 (~13.6-fold), IL-6 (~1.3-fold), and IL-1β (~2.1-fold). Upon pre-treatment with dexamethasone [1 µM] for 1 h, mRNA levels in DDCs were significantly decreased to ~1.6-fold for IL-8 (8.5-fold decrease), to ~0.3-fold (4.3-fold decrease) for IL-6, and to ~0.2-fold (10.5-fold decrease) for IL-1β. Similarly, pre-treatment with PsA-D [20 µM] for 1 h resulted in significantly lower mRNA levels for IL-8 (~2.0-fold; 6–8-fold decrease), IL-6 (~0.6-fold; 2.2-fold decrease), and IL-1β (~0.4-fold; 5.3-fold decrease).

Furthermore, upon NiSO_4_ [380 µM] exposure of DDCs for 6 h, the mRNA expression levels of COX-2, an enzyme integral to inflammatory prostaglandin synthesis, were upregulated 1.9-fold. Following pre-treatment with dexamethasone [1 µM] or PsA-D [20 µM] for 1 h, mRNA levels were reduced to ~1.3-fold (1.5-fold decrease) and 1.5-fold (1.3-fold decrease), respectively. 

The mRNA levels for NLRP3, a key inflammasome regulator, showed a modest increase in DDC surrogates following NiSO_4_ [380 µM] exposure for 6 h. Pre-treatment with dexamethasone [1 µM] for 1 h resulted in a 1.2-fold decrease in the mRNA levels for NLRP3. In comparison, the mRNA levels of NLRP3 remained unchanged in DDCs after PsA-D [20 µM] pre-treatment for 1 h. 

To investigate the anti-inflammatory impact of PsA-D in human skin with incorporated DDCs, we utilized our previously established engineered model, in which THP-1-derived DDC surrogates are co-seeded with primary human keratinocytes onto fibroblast-containing dermal equivalents [42]. Following 10 days of air–liquid interface (ALI) cultivation, the models achieved complete epidermal differentiation, forming all characteristic layers of native human skin, including a well-developed stratum corneum (Figure 5A,B). On day 10, the skin models were topically pre-treated with either dexamethasone [1 µM] or PsA-D [30 µM] for 1 h, followed by a 6 h exposure of NiSO_4_ [380 µM]. Subsequent mechanical and enzymatic dissociation was performed to enable downstream analysis of mRNA expression levels.

Consistent with the trends observed in isolated DDC surrogates (Figure 5C, Table 1), topical exposure of the DDC-containing skin model to NiSO_4_ [380 µM for 6 h] (Figure 5D, Table 2) led to increased mRNA expression of DC activation markers (CD54, CD86) and inflammatory mediators such as COX-2 and NLRP3. 

Specifically, upon topical treatment with NiSO_4_ [380 µM for 6 h], CD54 and CD86 mRNA levels increased by ~1.5-fold and ~1.2-fold, respectively. Dexamethasone [1 µM] pre-treatment for 1 h reduced CD54 and CD86 expression by ~1.9-fold and ~1.7-fold, respectively, whereas PsA-D pre-treatment resulted in reductions of 1.7-fold for CD54 and 1.5-fold for CD86. 

Furthermore, topical treatment of the DDC-skin model with NiSO_4_ [380 µM for 6 h] also significantly increased the expression of pro-inflammatory cytokines. Namely, IL-8 mRNA levels were elevated ~3.0-fold, but were suppressed to ~0.3-fold (10-fold decrease) following dexamethasone [1 µM] pre-treatment and to ~1.4-fold (2.1-fold decrease) with PsA-D [20 µM] pre-treatment for 1 h. Similarly, IL-6 mRNA expression, which increased significantly (~2.8-fold) after topical exposure of the DDC model to NiSO_4_ [380 µM], was attenuated to ~0.2-fold (14-fold decrease) and to ~1.1-fold (~2.6-fold decrease) with dexamethasone [1 µM] and PsA-D [20 µM] pre-treatment, respectively. IL-1β mRNA levels increased to ~2.8-fold following topical NiSO_4_ [380 µM] exposure for 6 h but were suppressed to ~0.5-fold (~5.6-fold decrease) with dexamethasone [ 1µM] and ~1.3-fold (~2.2-fold decrease) with PsA-D [20 µM] pre-treatment. 

The mRNA levels of COX-2 were significantly elevated (~3.5-fold) upon topical NiSO_4_ exposure but were reduced to ~0.1-fold (35-fold decrease) with dexamethasone [1 µM] and suppressed to ~1.0-fold (3.20-fold decrease) following topical PsA-D [20 µM] pre-treatment for 1 h. Lastly, NLRP3 mRNA levels, which increased ~1.6-fold upon topical NiSO_4_ treatment for 6 h, were downregulated to ~0.9-fold (~1.8-fold decrease) when dexamethasone [1 µM] and to ~0.8-fold (~2.1-fold decrease) when PsA-D was topically applied 1 h before NiSO_4_ [380 µM] exposure (6 h).

These findings indicate that both dexamethasone and PsA-D effectively mitigate NiSO_4_-induced inflammatory responses in the DDC surrogate and full-thickness skin models, while dexamethasone demonstrates a more pronounced suppressive effect in general.

## 3. Discussion

In this study, we explored the anti-inflammatory effects of PsA-D on NiSO_4_-induced immune activation using isolated dendritic cell (DDC) surrogates and a human engineered skin model with incorporated DDCs. Our findings revealed that PsA-D effectively suppressed key inflammatory markers and pathways associated with allergic contact dermatitis (ACD), demonstrating comparable mechanisms of action to dexamethasone, a benchmark corticosteroid—both in isolated dendritic cells (DDCs) and in a reconstructed skin model following their integration.

While pseudopterosin A (PsA) has been previously recognized for inhibiting neutrophil degranulation and macrophage activation [11,43], its impact on dendritic cell function is unexplored. Here, we show that PsA-D effectively attenuated NiSO_4_-induced upregulation of DC activation markers CD54 and CD86, both in isolated DDC surrogates and in our full-thickness DDC-skin model, suggesting that PsA-D can modulate early immune activation, a crucial step in ACD pathogenesis. 

Previous studies have indicated that pseudopterosins might modulate the immune response through modulation of key signaling pathways such as NF-κB in various cellular systems. In fact, it was shown that PsA-D attenuated NF-κB activation induced by lipopolysaccharide (LPS) and tumor necrosis factor-alpha (TNF-α) in both the human monocytic THP-1 cell line and the MDA-MB-231 triple-negative breast cancer (TNBC) cell line. This effect was mediated by stabilizing IκBα and inhibiting phosphorylation of the NF-κB subunit p65, resulting in reduced expression of pro-inflammatory cytokines such as TNF-α, IL-6, and MCP-1 [12]. Furthermore, PsA-D was shown to induce translocation of the glucocorticoid receptor alpha (GRα), with GRα knockdown abolishing its anti-inflammatory effects, suggesting that PsA-D leverages a glucocorticoid receptor-mediated mechanism similar to synthetic corticosteroids [13].

Building on these insights, our study identified the inhibition of NF-κB signaling as a strong mechanism underlying PsA-D’s anti-inflammatory effects in DDC surrogates. PsA-D effectively prevented NiSO_4_-induced NF-κB activation by stabilizing IκBα. Notably, PsA-D exhibited stronger inhibitory effects in the NiSO4-induced degradation of IkBa than dexamethasone. Thus, PsA-D appeared to exert a more targeted effect on NF-κB signaling, hinting at a more selective anti-inflammatory mechanism than dexamethasone. 

Furthermore, pre-treatment of DDC surrogates with PsA-D significantly reduced the secretion of key inflammatory cytokines, including IL-8, IL-6, and IL-1β in a similar manner as dexamethasone, underscoring the potential of PsA-D to mimic corticosteroid efficacy while possibly offering a more targeted and safer therapeutic profile.

A major strength of this study lies in the validation of PsA-D’s effects after topical administration in an immune-competent skin model comprising human fibroblasts, keratinocytes, and DDC surrogates, thereby providing a physiologically relevant platform for assessing skin inflammation. Topical pre-treatment with PsA-D for 1 h significantly reduced NiSO_4_-induced expression of inflammatory markers, including DC activation markers CD54 and CD86 and inflammatory cytokines IL-8, IL-6, and IL-1β as well as inflammasome-related genes such as COX-2 and NLRP3. 

COX-2 is involved in the conversion of arachidonic acid to prostaglandins, which are lipid compounds that mediate inflammation and pain [44,45]. Inhibition of sensitizer-induced COX-2 upregulation could therefore reduce inflammation, alleviate pain, and mitigate other associated cutaneous inflammatory responses. Notably, as early as 1982, Look et al. demonstrated that the topical application of PsA could more effectively counteract phorbol myristate acetate-induced inflammation compared to the classical COX inhibitor, indomethacin [3]. In fact, several studies have identified the arachidonic acid (AA) pathway as a key mechanism underlying the anti-inflammatory effects of pseudopterosins [3,11,40]. Specifically, the administration of PsA has been shown to inhibit the production of two eicosanoids, prostaglandin E2 (PGE2) and leukotriene C4 (LTC4), in murine peritoneal macrophages, suggesting an influence on the COX and LOX enzymes involved in the AA pathway [3,11].

NLRP3 is a key component of the inflammasome that plays a crucial role in regulating inflammatory responses. Its activation leads to the release of pro-inflammatory cytokines such as IL-1β and IL-18, which drive immune cell recruitment and amplify the inflammatory cascade [25]. The observed suppression of NiSO_4_-induced NLRP3 expression after topical administration of PsA-D in the skin model offers a promising strategy to modulate this response, reducing the activation of DDCs and potentially other immune cells, thereby limiting the inflammatory cycle that contributes to the development and progression of skin sensitization.

Notably, the effects of NiSO_4_, as well as the anti-inflammatory effects of PsA-D and dexamethasone, appear to be more pronounced in most genes in the isolated DDC surrogates. However, it is crucial to consider that the isolated DDC surrogates represent a single cell type, whereas the full-thickness skin model consists of three distinct cell types and a matrix. After 10 days in the ALI Phase, DDC surrogates account for only 2–4% of the total cell population in the full-thickness skin model, representing the in vivo situation in native human skin where dendritic cells make up approximately 1–3% of the skin cell population [32]. This lower proportion of DDC surrogates in the full-thickness model likely contributes to the different effects.

Interestingly, the effects of NiSO_4_, along with the anti-inflammatory effects of PsA-D and dexamethasone, appear to be more pronounced in the skin model for COX-2 and NLRP3 expression. These observations may be attributed to the high prevalence of keratinocytes, which are known to activate the NLRP3 inflammasome in response to sensitizers, leading to the release of pro-inflammatory cytokines such as interleukin (IL)-1 and IL-8. Although no studies to date have specifically investigated the impact of sensitizers on COX-2 expression in keratinocytes, previous research has shown that exposure to UVB radiation [46] or irritant chemicals (such as carrageenan) [47] induces the upregulation of COX-2 in human keratinocytes, resulting in increased production of prostaglandin E2 (PGE2).

In summary, our findings indicate that both PsA-D and dexamethasone effectively attenuate NiSO_4_-induced inflammatory responses in isolated DDC surrogates and a DDC-skin model. While dexamethasone exerts a slightly stronger immunosuppressive effect and remains the standard treatment for allergic contact dermatitis (ACD) [35], its long-term use is limited by adverse effects such as skin atrophy, barrier dysfunction, and systemic immunosuppression [36,37]. PsA-D, though slightly less efficacious, demonstrates substantial anti-inflammatory activity and may represent a more gentle therapeutic alternative. However, its high lipophilicity and poor aqueous solubility contribute to low bioavailability, posing challenges for its broader pharmacological application [17]. Conversely, PsA-D’s lipophilic nature may be advantageous for topical use by enhancing stratum corneum penetration and promoting epidermal retention, thereby supporting localized therapeutic effects while minimizing systemic exposure. However, its pronounced lipophilicity may also limit deeper dermal penetration [48], which might explain the slightly less anti-inflammatory effects of PsA-D on our tissue-integrated DDC surrogates. Formulation strategies such as the use of cyclodextrins—which have been shown to enhance PsA potency up to 200-fold in HUVEC proliferation assays [17]—may help overcome these limitations. Future studies need to explore the long-term effects of PsA-D on skin homeostasis, as well as its pharmacokinetics and alternative formulation strategies for topical applications.

In conclusion, this study provides compelling evidence that PsA-D exerts significant anti-inflammatory effects by modulating dendritic cell activation by inhibiting NF-κB signaling, and suppressing inflammatory cytokine secretion. The validation of these effects in a full-thickness immune-competent skin model further supports its promising therapeutic potential for ACD and other inflammatory skin disorders. 

## 4. Materials and Methods

### 4.1. Preparation of the PsA-D 

Specimens of *A. elisabethae* were harvested from South Bimini Island, the Bahamas, then air-dried before being extracted with a 1:1 mixture of ethyl acetate and methanol over 48 h. The resulting crude extract was fractionated using silica gel chromatography with a hexane–ethyl acetate gradient, yielding a mixture containing PsA through PsD compounds. Liquid chromatography–mass spectrometry (LC-MS) analysis indicated that the composition ratio of PsA to PsB, PsC, and PsD was 85:5:5:5.

### 4.2. Cell Line Cultivation

The human monocytic leukemia cell line THP-1 (#TIB202, LOT:70025047) was obtained from ATCC (Manassas, VA, USA). THP-1 cells were cultured in T75 flasks (Greiner, #658195, Frickenhausen, Germany) containing 20 mL of RPMI medium (Gibco, #22400089, Grand Island, NY, USA) supplemented with 10% FBS (Gibco, #10270-106), 50 U/mL penicillin-streptomycin (PenStrep) (Gibco, #15140122), and 0.05 mM 2-mercaptoethanol (Gibco, #21985023). The cells were maintained in a humidified incubator at 37 °C with 5% CO_2_, with cell density kept between 1 × 10^5^ cells/mL and 5 × 10^5^ cells/mL.

### 4.3. Generation of DDC Surogates

Dermal dendritic cell (DDC) surrogates were generated following our previously published protocol [49]. Briefly, a total of 1 × 10^6^ THP-1 cells were seeded into a T25 flask containing 5 mL of RPMI-1640 medium (Gibco, #22400089) supplemented with 10% FBS (Gibco, #10270-106), 50 U/mL penicillin–streptomycin (Gibco, #15140122), and 0.05 mM 2-mercaptoethanol (Gibco, #21985023). Differentiation was induced by adding 1500 IU/mL rhGM-CSF (ImmunoTools, #11343125, Friesoythe, Germany) and 1500 IU/mL rhIL-4 (ImmunoTools, #11340045), with a medium change performed on day 3. The cells were incubated for a total of 5 days at 37 °C and 5% CO_2_.

### 4.4. Determination of Cytotoxicity

Amounts of 6 × 10^4^ DDC surrogates were seeded in a 96-well plate (Sarstedt, (#83.3924, Nümbrecht, Germany). Cells were treated with dexamethasone (Peprotech, #5000222, Hamburg, Germany) or PsA-D in the following concentrations: 70 µM, 50 µM, 40 µM, 35 µM, 30 µM, 20 µM, 10 µM, 1 µM or the respective DMSO control [1%] for 24 h, respectively. Finally, PrestoBlue^TM^ reagent (10 μL/well) (Invitrogen, P50201, Waltham, MA, USA) was added, and a blank control was prepared. After 1 h of incubation at 37 °C, 5% CO_2_, cell viability was assessed with a Tecan Spark microplate reader (Tecan Gropup AG, Männedorf, Switzerland). 

Skin models were treated topically with either 30 µM of PsA-D or the respective solvent control DMSO [0.03%] for 24 h. To determine cell viability, skin models were placed in 900 µL keratinocyte medium (Phenion, #K CM-250, Düsseldorf, Germany) and 100 µL PrestoBlue^TM^ reagent was added. After incubation for 1 h at 37 °C, 5% CO_2_, the cell viability was determined with a Tecan Spark microplate reader (Männedorf, Switzerland).

### 4.5. Sensitization Assays for Surface Marker Detection

DDC surrogates were seeded at a density of 1 × 10⁶ cells per well in a 24-well plate containing 1 mL of RPMI-1640 medium supplemented with 10% FBS, 50 U/mL penicillin–streptomycin, and 50 μM 2-mercaptoethanol. Cells were pre-treated with dexamethasone [1 µM] (Peprotech, #5000222, Cranbury, NJ, USA) or PsA-D [20 µM] for 1 h. Afterwards, cells were treated with NiSO_4_ (380 μM) (Sigma-Aldrich, #227676, Darmstadt, Germany). After 24 h, cells were harvested, washed three times in autoMACS running buffer (Miltenyi Biotec, #130-091-221, Gladbach, Germany), and transferred to 96-well plates (2 × 10^5^ cells per antibody panel). Cells were stained with the following antibodies (diluted 1:50) for 10 min at 4 °C in the dark: REA Control (S)-APC (Miltenyi Biotec, #130-113-434), CD54-APC (Miltenyi Biotec, #130-121-342), and CD86-APC (Miltenyi Biotec, #130-116-161). After staining, cells were washed three times with autoMACS running buffer and stained with DAPI (Sigma-Aldrich, #D9542, Darmstadt, Germany) to exclude dead cells and assess viability. Surface marker expression was analyzed via flow cytometry.

### 4.6. Western Blot Analysis

Amounts of 1 × 10⁶ cells were plated in a 24-well plate with 1 mL RPMI supplemented with 10% FBS, 50 U/mL penicillin–streptomycin, and 50 μM 2-mercaptoethanol. Cells were pre-treated with dexamethasone [1 µM] (Peprotech, #5000222) or PsA-D [20 µM] for 1 h, followed by NiSO_4_ [500 µM] (Sigma-Aldrich, #227676) treatment for 1 h. Following treatment, cells were harvested, washed with PBS, and lysed using RIPA buffer containing protease (Roche, #11836170001, Basel, Switzerland) and phosphatase inhibitors (Roche, #04906845001). Protein concentrations were quantified with a BCA protein assay kit (Thermo Scientific, #23227, Waltham, MA, USA). For Western blot analysis, 20 μg of protein lysate was mixed with Laemmli buffer (Bio-Rad, #1610747, Hercules, CA, USA). SDS page and Western blotting was performed as published previously [42]. The following primary antibodies were used: IκBα (Cell Signaling Technology, #9242S, Danversn, MA, USA) and vinculin (Cell Signaling Technology, #13901S). The membrane was incubated with the appropriate horseradish peroxidase-conjugated secondary antibody (Goat anti-Rabbit (H+L) (Thermo Fisher Scientific, #31460, Waltham, MA, USA). Antibody binding was visualized using the SuperSignal West Pico Plus substrate kit (Thermo Fisher Scientific, #34577) and imaged with a ChemStudio Imager (Analytik Jena, #849-97-0928-04, Jena, Germany).

### 4.7. Cytokine Secretion

DDC surrogates were seeded at a density of 1 × 10⁶ cells per well in a 24-well plate containing 1 mL of RPMI-1640 medium supplemented with 10% FBS, 50 U/mL penicillin–streptomycin, and 50 μM 2-mercaptoethanol. Cells were pre-treated with dexamethasone [1 µM] (Peprotech, #5000222) or PsA-D [20 µM] for 1 h. Afterwards, cells were treated with NiSO_4_ (380 μM) (Sigma-Aldrich, #227676). After 24 h, supernatants were collected for cytokine analysis. The secretion of inflammatory cytokines was measured using the Cytometric Bead Array Human Inflammatory Cytokines Kit (BD, #551811, Franklin Lakes, NJ, USA) according to the manufacturer’s instructions, and data acquisition was performed using the CytoFlex (B5-R3-V5) from Beckman Coulter (Brea, CA, USA). Data analysis was carried out with the CBA Analysis Software (Version 1.1.14) (BD Biosciences, Franklin Lakes, NJ, USA).

### 4.8. Incorporation of DDCs into Full-Thickness Skin Models

DDC-containing skin models were generated as published in our previous study [42]. Briefly, primary human foreskin keratinocytes (Phenion, #hK P1) from juvenile donors were grown in a feeder cell (Phenion, #hFeeder)-supported cultivation. Keratinocytes at passage 2 (5 × 10^5^) were then co-seeded with 1 × 10⁶ DDC surrogates (1:2 ratio) in 1 mL keratinocyte medium (Phenion, #K CM-250) onto dermis models (in inserts) consisting of a solid and porous collagen matrix along with primary human foreskin fibroblasts (obtained from Henkel AG & Co. KGaA, Düsseldorf, Germany). The skin models underwent a 48 h submerged phase before being transferred to an air–liquid interface, where they were cultured with Air–Liquid Interface Culture Medium (Phenion, #ALI CM HC-250, without hydrocortisone) for 10 days.

### 4.9. Cryosectioning and Immunofluorescence Staining

Cryosectioning and immunofluorescence staining were performed following our previously published protocols [42]. Briefly, skin models were embedded, frozen, and cryosectioned into 7 µm slices. After fixation in cold acetone, tissue slides were blocked with 10% normal goat serum (Invitrogen, #50062Z). The primary antibodies, cytokeratin 5 (OriGene, #DM361; dilution 1:75, Rockville, MD, USA) and CD45-VioBright R667 (Miltenyi, #130-110-779; dilution 1:50), were applied at 4 °C overnight. Afterward, secondary antibody staining was carried out using Alexa Fluor 488 (Invitrogen, #A11017; dilution 1:200) and DAPI (10 µg/mL) (Sigma, #D9542). The stained sections were mounted with Tissue Fluorescence mounting medium (Agilent, #S3023, Santa Clara, CA, USA) and were visualized using confocal spinning disk microscopy (CQ1, Yokogawa, Musashino, Tokyo, Japan).

### 4.10. Skin Model Dissociation and RNA Isolation

For RNA isolation, skin models were minced into small pieces. RNA was isolated using the RNeasy Mini Kit (Qiagen, #74104, Düsseldorf, Germany), the DNase Kit (Qiagen, #79254), and proteinase K (Qiagen, #19133). Enzymatic dissociation was carried out by incubating the minced tissue samples with RLT buffer from the RNeasy Kit at 20 °C for 45 min, followed by an additional incubation with proteinase K for 30 min at 55 °C with shaking at 400 RPM. After centrifugation, the supernatant was combined with 0.7× *g* its volume of 98% ethanol and passed through a RNeasy spin column. A single washing step with RW1 buffer was performed before treating the samples with RNase/RDD solution (10 μL + 70 μL) from the DNase Kit for at least 15 min. Subsequent steps were performed according to the manufacturer’s instructions for the RNeasy Mini Kit.

### 4.11. RT Quantitative PCR (RT-qPCR)

cDNA synthesis and qPCR were performed as described in former studies [42,50]. Briefly, reverse transcription was carried out with the QuantiTect Reverse Transcription Kit (Qiagen, #205311), using 1 μg of RNA for cDNA synthesis. Quantitative real-time PCR (qPCR) was conducted in triplicate for each sample, with 50 ng of cDNA per reaction, using the Luna Universal qPCR Master Mix (NEB, #M3003L, Ipswich, MA, USA). The specific primers used were as follows: GAPDH (forward: 5′-TGCACCACCAACTGCTTAGC-3′; reverse: 5′-GGCATGGACTGTGGTCATGAG-3′), CD54 (forward: 5′-AGCGGCTGACGTGTGCAGTAAT-3′; reverse: 5′-TCTGAGACCTCTGGCTTCGTCA-3′), CD86 (forward: 5′-CCATCAGTCTGTCTGTTTCATTCC-3′; reverse: 5′-GCTGTAATCCAAGGAATGTGGTC-3′), IL-6 (forward: 5′GGCACTGGCAGAAAACAACC–3′; reverse: 5′-GCAAGTCTCCTCATTGAATCC-3′), IL-8 (forward: 5′-ACTGAGAGTGATTGAGAGTGGAC-3′; reverse: 5′-AACCCTCTGCACCCAGTTTTC-3′); IL-1β (forward: 5′-GCACGATGCACCTGTACGAT-3′; reverse: 5′-CACCAAGCTTTTTTGCTGTGAGT-3′); COX-2 (forward. 5′-CGG TGA AAC TCT GGC TAG ACA G-3′; reverse: 5′-GCAAACCGTAGATGCTCAGGGA-3′), and NLRP3 (forward: 5′-GGACTGAAGCACCTGTTGTGCA-3′; reverse: 5′-TCCTGAGTCTCCCAAGGCATTC-3′).

## Figures and Tables

**Table 1:** mRNA expression levels in DDC surrogates upon exposure to NiSO_4_ [380 µM] for 6 h or pre-treatment with dexamethasone [1 µM] or pseudopterosin A-D [20 µM] for 1 h compared to the solvent control.

Gene	Ctrl [0.3% DMSO]	NiSO_4_ [380 µM]	Dex [1 µM] + NiSO_4_ [380 µM]	PsA-D [20 µM] + NiSO_4_ [380 µM]
*CD54*	1	~1.9	~0.9 (~2.1-fold decrease ****)	~0.9 (~2.1-fold decrease ****)
*CD86*	1	~1.1	~0.4 (~2.8-fold decrease ****)	~0.8 (~1.4-fold decrease **)
*IL-8*	1	~13.6	~1.6 (~8.5-fold decrease ****)	~2.0 (~6.8-fold decrease ****)
*IL-6*	1	~1.3	~0.3 (~4.3-fold decrease ***)	~0.6 (~2.2-fold decrease **)
*IL-1β*	1	~2.1	~0.2 (~10.5-fold decrease ****)	~0.4 (~5.3-fold decrease ****)
*COX-2*	1	~1.9	~1.3 (~1.5-fold decrease)	~1.5 (~1.3-fold decrease)
*NLRP3*	1	~1.1	~0.9 (~1.2-fold decrease)	~1.1 (No change)

Statistical significance of the fold decrease compared to NiSO_4_ exposure alone is indicated for *n* = 3 independent experiments with ** = *p* ≤ 0.01, *** = *p* ≤ 0.001, and **** = *p* ≤ 0.0001.

**Table 2:** mRNA expression levels in DDC full-thickness skin models upon topical exposure to NiSO_4_ [380 µM] or pre-treatment with dexamethasone [1 µM] or pseudopterosin A-D [20 µM] compared to the solvent control.

Gene	Ctrl [0.3% DMSO]	NiSO_4_ [380 µM]	Dex [1 µM] + NiSO_4_ [380 µM]	PsA-D [20 µM] + NiSO_4_ [380 µM]
*CD54*	1	~1.5	~0.8 (~1.9-fold decrease **)	~0.9 (~1.7-fold decrease *)
*CD86*	1	~1.2	~0.7 (~1.7-fold decrease **)	~0.8 (~1.5-fold decrease **)
*IL-8*	1	~3.0	~0.3 (~10-fold decrease ***)	~1.4 (~2.1-fold decrease *)
*IL-6*	1	~2.8	~0.2 (~14-fold decrease ****)	~1.1 (~2.6-fold decrease ****)
*IL-1β*	1	~2.8	~0.5 (~5.6-fold decrease ****)	~1.3 (~2.2-fold decrease **)
*COX-2*	1	~3.5	~0.1 (~35-fold decrease ****)	~1.0 (~3.5-fold decrease ****)
*NLRP3*	1	~1.6	~0.9 (~1.8-fold decrease **)	~0.8 (~2.1-fold decrease **)

Statistical significance of the fold decrease compared to NiSO_4_ exposure alone is indicated for *n* = 3 independent experiments with * = *p* ≤ 0.05, ** = *p* ≤ 0.01, *** = *p* ≤ 0.001, and **** = *p* ≤ 0.0001.

## Data Availability

The original contributions presented in this study are included in the article. Further inquiries can be directed to the corresponding author.
